# Superior survival for breast-conserving therapy over mastectomy in patients with breast cancer: A population-based SEER database analysis across 30 years

**DOI:** 10.3389/fonc.2022.1032063

**Published:** 2023-01-04

**Authors:** Shanbao Ke, Wei Wang, Baiyu Li, Xiao Feng, Danfang Yan, Jianbo Liu

**Affiliations:** ^1^ Department of Oncology, Henan Provincial People's Hospital, Zhengzhou University People's Hospital, Zhengzhou, China; ^2^ Department of Radiation Oncology, The First Affiliated Hospital, College of Medicine, Zhejiang University, Hangzhou, China

**Keywords:** breast cancer, recurrence, cancer-specific survival, competing risk, propensity score weighting, SEER

## Abstract

**Introduction:**

It has been believed that breast-conserving therapy (lumpectomy plus adjuvant radiation, Lum + RT) and mastectomy without radiation (Mast + NoRT) have equivalent survival outcomes. However, there is a need to re-evaluate the role of lumpectomy plus adjuvant radiation due to changed breast cancer management over time. This study aimed to conduct a population-based study that compare long-term oncologic survival outcomes after Lum + RT vs Mast + NoRT.

**Methods:**

The Surveillance, Epidemiology and End Results database was used to identify female breast cancer patients with a primary localized breast cancer diagnosis from 1988 to 2018. The standardized incidence/mortality ratio (SIR/SMR) for breast cancer recurrence (BCR) and breast cancer-specific death (BSD) was estimated by the SEER*Stat program. Cumulative incidences of BCR and BSD were assessed using Gray’s method. We evaluated the effects of Lum + RT vs. Mast + NoRT on breast cancer recurrence-free survival (BRFS) and breast cancer-specific survival (BCSS). Fine-Gray competing risk model analyses, propensity score-adjusted Kaplan-Meier analyses and Cox proportional hazards model analyses were applied.

**Results:**

A total of 205,788 women were included in the study. Patients who underwent Lum + RT had higher SIR of BCR (4.14 [95% confidence interval, CI: 3.94-4.34] vs. 1.11 [95% CI: 1.07-1.14]) and lower SMR (9.89 [95% CI: 9.71-10.08] vs. 17.07 [95% CI: 16.82-17.33]) than patients who underwent Mast + NoRT. Lum + RT was associated with higher competing risk of BCR (adjusted hazard ratio [HR]: 1.996, 95% CI: 1.925-2.069, p < 0.001) and lower competing risk of BSD when compared to Mast + RT (adjusted HR: 0.584, 95% CI: 0.572-0.597, p < 0.001). Multivariate Cox regression analysis revealed similar results (adjusted HR after PSW for BRFS: 1.792, 95% CI 1.716-1.871, p < 0.001; adjusted HR after PSW for BCSS: 0.706, 95% CI 0.688-0.725, p < 0.001). These findings persisted in the sensitivity and subgroup analyses.

**Discussion:**

The present study further confirmed superior long-term survival with lumpectomy plus adjuvant radiation over mastectomy independent of patient characteristics including age, race, time period, historic subtype, tumor size, historic grade and stage, indicating that this benefit may result from the treatment itself.

## Introduction

Breast-conserving therapy (lumpectomy plus adjuvant radiotherapy) has become the primary treatment option for patients with early-stage breast cancer since a National Institutes of Health (NIH) Consensus Statement released in 1991 recommended that breast conserving therapy (BCT) offered equivalent survival compared with mastectomy (1). The use of BCT has increased rapidly during the last 2–3 decades as it provides superior cosmetic and quality-of-life outcomes (2–4).

However, mastectomy has recently re-emerged to play an essential role in breast cancer treatment for limited radiotherapy resources, patient preference, improvements in breast reconstruction techniques, and increased use of BRCA testing (5–9). In addition, radiotherapy after breast-conserving surgery has raised concerns about an increased risk of locoregional recurrence (10–14).

Some clinical trials and observational studies have compared oncological outcomes of mastectomy versus BCT (13, 15–25). and have not yielded conclusive results. Several reports also pointed out the need to re-evaluate the role of BCT because of recent advances in treating breast cancer with radiotherapy (26–28). Moreover, most of these studies used the Kaplan-Meier (KM) survival method, which is generally meant to describe time to a single type of event and is often unsuitable when comparing cause-specific outcomes in patients with multiple potential outcomes (29–31). The competing risk methodology may be more suitable to adjust for the influence of competing events because estimating the incidence of events of interest in the competing risk model is conditional on the composite event rate of all events of interest and those competing events (32–34). A recent study applied this method to data reported by the Early Breast Cancer Trialists Collaborative Group (EBCTCG), which confirmed that KM-based methods led to biased risk estimates in early breast cancer studies, especially for uncommon outcomes such as local recurrence (35).

In the present study, we adopted the competing risk method in population-based data from Surveillance, Epidemiology, and End Results (SEER). We aimed to compare the long-term oncological outcomes including breast cancer recurrence-free survival (BRFS) and breast cancer-specific survival (BCSS) between lumpectomy plus adjuvant radiation (Lum + RT) and mastectomy without radiation (Mast + NoRT). Furthermore, adjusted KM estimates and Cox proportional hazards models using a propensity score weighting (PSW) method were applied to balance the observed covariates between patients who underwent Lum + RT and those who underwent Mast + NoRT.

## Materials and methods

### Data source and study population

Data was obtained from the SEER database, the largest publicly available cancer dataset, covering approximately 47.9 percent of the U.S. population. The exact dataset we used for this study was SEER Program Research Data (1988–2018), National Cancer Institute, DCCPS, Surveillance Research Program, Surveillance Systems Branch, based on the November 2021 submission (36).

We used SEER* Stat 8.4.0 software to retrieve demographic and clinical information of female patients with breast cancers diagnosed between 1988 and 2018 from the SEER database. Patients diagnosed before 1988 were not included in this study because data regarding lymph node involvement were not uniformly recorded in the SEER database before this point. The following inclusion criteria were applied (1): ICD WHO site recode of ‘breast’ (2); age 18 years or greater at the time of diagnosis (3); primary site of breast cancer (4); localized and regional SEER historic stage. We excluded patients of unknown race or stage, or diagnosed by death certificates or autopsy.

### Treatment modalities for breast cancer

Patients treated with lumpectomy and postoperative radiation were included in the Lum + RT group. Lumpectomy was identified by site-specific surgery codes 10–28 or surgery of primary site codes 20-24. The Mast + NoRT group was composed of patients who underwent mastectomy alone. Mastectomy was identified by site-specific surgery codes 30-90 or surgery of primary site codes 30-90. We excluded patients who received other types of treatment interventions.

### Study variables and outcomes

The following demographic and clinical variables were extracted from the SEER database: age at diagnosis, race, year of diagnosis, historic subtype, tumor size, historic grade, chemotherapy, ER/PR status, HER2 status. Tumor staging was derived according to the American Joint Committee on Cancer (AJCC) 7th edition staging systems using data on tumor size, lymph node involvement, and distant metastasis (37). Laterality was categorized into three groups according to the lateralities of the primary breast cancer and the recurrence breast cancer (ipsilateral, contralateral, and bilateral/unspecified).

The primary endpoints of our study were BRFS and BCSS. Follow-up began 12 months after breast cancer surgery, ensuring patients who had undergone adjuvant radiotherapy or chemotherapy. BRFS was defined as the period from the date of diagnosis to the date of any breast cancer recurrence, and BCSS was measured from the date of diagnosis to the date of death from breast cancer. Patients with second primary cancers or who died due to causes other than breast cancer or with an unknown cause of death were excluded from the analysis.

### Statistical analysis

Patients’ demographic and tumor characteristics were compared between the treatment groups. Comparisons of categorical variables were performed using the Chi-square test, and continuous variables were compared using Student’s t test. The standardized incidence ratio (SIR) of recurrence and breast cancer-specific standardized mortality ratio (SMR) among breast cancer survivors was estimated by the SEER*Stat MP-SIR session. The SIR/SMR was calculated by dividing the observed incidence of recurrence or observed cancer-specific mortality by the expected incidence of recurrence or expected cancer-specific mortality [observed/expected (O/E) ratio] in the U.S. general population. Stratified SIR/SMRs were calculated based on attained age, calendar year, and latency (interval time from primary breast cancer diagnosis to recurrence or cancer-specific death). The cumulative incidence function (CIF) was adopted to estimate the probability of breast cancer recurrence (BCR) and breast cancer-specific death (BSD) by the Gray’s subdistribution hazard method (38). Univariable and multivariable competing risk regression models by Fine and Gray were used to assess the risk of BRFS and BCSS in patients who received different treatment modalities (39). A new PSW approach based on overlap weight (OW) was applied to balance observed covariates between the treatment groups. The popular inverse probability weighting method is limited by biased estimates induced by extreme weights when the propensity score distributions between the treatment groups lack overlap (40, 41). The OW method overcomes essential limitations of traditional weighting approaches by emphasizing the target population with the most overlap (42, 43). Survival function estimations for BCR and BSD were adopted using KM estimates and the log-rank test. Univariate and multivariate Cox proportional hazards models were performed to analyze the association between patient treatments with BRFS and BCSS before and after PSW. Recurrence laterality was not included as only patients with BCR had this information, nor were ER/PR and HER2 status included because these data were not routinely reported to SEER registries prior to 1990 or 2010. Sensitivity analyses were conducted in patients with different ER/PR statuses. Stratified analyses were also carried out to examine the impact of covariates. Hazard ratios (HRs) and 95% CIs adjusted by the PSW method were estimated separately for these subgroups. All p values were two-sided and p < 0.05 was considered statistically significant. Data analysis was performed using R software (version 4.1.2).

## Results

### Patient characteristics

Within the SEER database, we identified 532,616 female patients with breast cancer diagnosed between 1988 and 2019. Among these patients, 235,101 were excluded. Consequently, a total of 205,788 patients were included in the final cohort. Detailed patient selection flowchart is given in **Figure S1**. The final study population consisted of 124,164 patients treated with Lum + RT and 81,624 patients treated with Mast + NoRT. The median age at diagnosis for all patients was 56 (48–66) years. The median follow-up duration was 114 months in total, and 112 and 116 months for the Lum + RT group and the Mast + NoRT group, respectively. Between 1988 and 1995, mastectomy without radiation was more common in female breast cancer patients. The use of lumpectomy plus adjuvant radiation became more frequent and had increased over the last two decades (1996 to 2018) (**Figure S2A**).

**Table 1** shows significant differences in the clinical characteristics of patients between the treatment groups. There were 10,913 (5.3%) breast cancer recurrences and 30,341 (14.7%) breast cancer-specific deaths in total. The overall median BRFS time was 115 months and the overall median BCSS time was 68 months. The median BRFS and BCSS time were longer for patients treated with Lum + RT than those treated with Mast + NoRT (118 vs. 108 months and 77 vs. 64 months, respectively) (**Figure S2B**).

**Table 1:** Baseline characteristics of 205,788 female breast cancer patients by treatment.

Variables		Lum + RT	Mast + NoRT	*P* value
		(N=124164)	(N=81624)	
Age				<0.001
	median (IQR)	57 (49-66)	55 (46-65)	
	18-44	14012 (11.3%)	14865 (18.2%)	
	45-59	53283 (42.9%)	34059 (41.7%)	
	≥60	56869 (45.8%)	32700 (40.1%)	
Race				<0.001
	White	100962 (81.3%)	65926 (80.8%)	
	Black	9032 (7.3%)	5826 (7.1%)	
	Other	14170 (11.4%)	9872 (12.1%)	
Year of Diagnosis				<0.001
	1988-1999	23699 (19.1%)	29817 (36.5%)	
	2000-2009	43225 (34.8%)	24425 (29.9%)	
	2010-2018	57240 (46.1%)	27382 (33.5%)	
Historic Subtype				<0.001
	Ductal	96754 (77.9%)	59423 (72.8%)	
	Lobular	15433 (12.4%)	13051 (16.0%)	
	Others	11977 (9.7%)	9150 (11.2%)	
Tumor Size				<0.001
	<1cm	32555 (26.2%)	14376 (17.6%)	
	1-2cm	53774 (43.3%)	25688 (31.5%)	
	2-3cm	23256 (18.7%)	18584 (22.8%)	
	≥3cm	12599 (10.1%)	18977 (23.2%)	
	Unknown	1980 (1.6%)	3999 (4.90%)	
Grade				<0.001
	I-II	81317 (65.5%)	42061 (51.5%)	
	III-IV	34267 (27.6%)	27938 (34.2%)	
	Unknown	8580 (6.91%)	11625 (14.2%)	
Stage				<0.001
	I	79088 (63.7%)	35882 (44.0%)	
	II	36468 (29.4%)	32485 (39.8%)	
	III	5633 (4.5%)	9363 (11.5%)	
	Unknown	2975 (2.4%)	3894 (4.8%)	
Chemotherapy			
	Yes	48633 (39.2%)	33759 (41.4%)	<0.001
	No	75531 (60.8%)	47865 (58.6%)	
ER/PR Status				<0.001
	ER-/PR-	17686 (14.2%)	13762 (16.9%)	
	ER-/PR+	1708 (1.4%)	1512 (1.9%)	
	ER+/PR-	12170 (9.8%)	8048 (9.9%)	
	ER+/PR+	85932 (69.2%)	45528 (55.8%)	
	Borderline/Unknown	4942 (4.0%)	6957 (8.5%)	
	Not available	1726 (1.4%)	5817 (7.1%)	
HER2 Status				<0.001
	Positive	6444 (5.2%)	4745 (5.8%)	
	Negative	48905 (39.4%)	21207 (26.0%)	
	Borderline/Unknown	1849 (1.5%)	1372 (1.7%)	
	Not available	66966 (53.9%)	54300 (66.5%)	
Status				<0.001
	Alive without BCR	105361 (84.9%)	59173 (72.5%)	
	BSD	11234 (9.0%)	11234 (23.4%)	
	BCR	7569 (6.1%)	7569 (4.1%)	
Recurrence laterality				<0.001
	Ipsilateral	4340 (57.2%)	3010 (89.1%)	
	Contralateral	3202 (42.2%)	315 (9.3%)	
	Bilateral/unspecified	44 (0.6%)	54 (1.6%)	

IQR interquartile range; BCR breast cancer recurrence; BSD breast cancer-specific death.

### Standardized incidence ratio of BCR and Standardized mortality ratio of BSD

**Table 2** presents the SIRs of BCR and the SMRs of BSD. The overall SIR of BCR for patients who received Lum + RT was 4.14 (95% CI: 3.94-4.34), and the overall SIR of BCR for patients who received Mast + NoRT was much lower (SIR, 1.11; 95% CI: 1.07-1.14). The same trends were observed in the SIRs stratified by attained age, calendar year, and latency. On the contrary, the SMRs of BCD for patients in the Lum + RT group were significantly lower than that for patients in the Mast + NoRT group (overall SMR: 9.89 [95% CI: 9.71-10.08] vs. 17.07 [95% CI: 16.82-17.33]), similar results were seen in subgroup analyses (**Table 2**).

**Table 2:** Standardized incidence ratios (SIRs) for breast cancer recurrence and standardized mortality ratios (SMRs) for breast cancer-specific death by treatment.

	SIR						SMR					
	Lum + RT			Mast + NoRT			Lum + RT			Mast + NoRT		
	Observed	Expected	O/E (95% CI)	Observed	Expected	O/E (95% CI)	Observed	Expected	O/E (95% CI)	Observed	Expected	O/E (95% CI)
Total	1,680	406	4.14 (3.94-4.34)	4,684	4,234.80	1.11 (1.07-1.14)	10,667	1078.2	9.89 (9.71-10.08)	17,596	1,030.50	17.07 (16.82-17.33)
Attained age												
18-44	411	55.4	7.42 (6.72-8.18)	263	58.9	4.46 (3.94-5.04)	776	6.6	117.77 (109.63-126.36)	1485	7.3	203.18 (192.97-213.78)
45-59	1,060	282.9	3.75 (3.53-3.98)	988	779.7	1.27 (1.19-1.35)	3,092	142.7	21.67 (20.91-22.44)	4,399	117.9	37.32 (36.23-38.44)
60+	209	67.7	3.09 (2.68-3.53)	3,433	3,396.10	1.01 (0.98-1.05)	6799	928.9	7.32 (7.15-7.5)	11,712	905.4	12.94 (12.7-13.17)
Calendar year												
1988-1999	177	35.4	5 (4.29-5.8)	1,202	964.1	1.25 (1.18-1.32)	1699	109.8	15.47 (14.74-16.22)	6,399	248.5	25.75 (25.12-26.39)
2000-2009	646	140.9	4.58 (4.24-4.95)	1,846	1,601.40	1.15 (1.1-1.21)	4334	392.2	11.05 (10.72-11.38)	6,610	403	16.4 (16.01-16.8)
2010-2018	857	229.7	3.73 (3.49-3.99)	1,636	1,669.20	0.98 (0.93-1.03)	4634	576.1	8.04 (7.81-8.28)	4,587	379	12.1 (11.76-12.46)
Latency												
1-10 years	818	193.2	4.23 (3.95-4.53)	2,796	2,581.50	1.08 (1.04-1.12)	7441	659.5	11.28 (11.03-11.54)	13,458	598.5	22.48 (22.11-22.87)
10-20 years	693	161.3	4.3 (3.98-4.63)	1,530	1,300.90	1.18 (1.12-1.24)	2779	353.6	7.86 (7.57-8.16)	3,490	333.9	10.45 (10.11-10.81)
20-30 years	169	51.5	3.28 (2.8-3.81)	358	352.4	1.02 (0.91-1.13)	447	65.1	6.87 (6.24-7.53)	648	98.1	6.6 (6.1-7.13)

CI, confidence interval.

### Cumulative incidence of BCR and BSD

**Figure 1:**
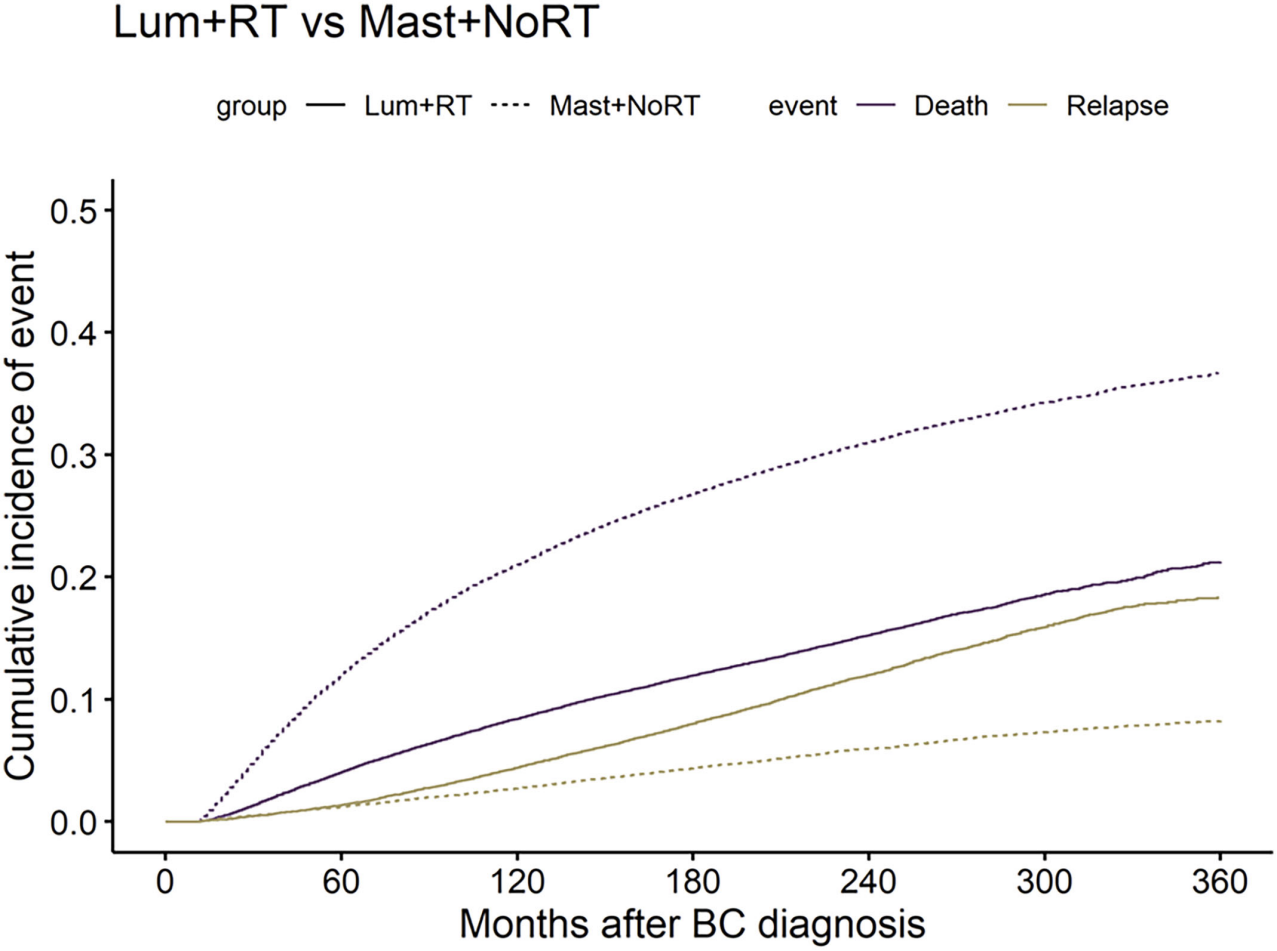
Comparisons of cumulative incidence of breast cancer recurrence (BCR) and breast cancer-specific death (BSD) between patients who received lumpectomy with adjuvant radiation (Lum + RT) and patients received mastectomy without radiation (Mast + NoRT). The solid lines represent Lum + RT, the dotted lines represent Mast + NoRT; The yellow lines represent BCR, the purple lines represent BSD. BC, breast cancer.

The 30-year CI of BCR was 18.44% in patients who received Lum + RT, while the incidence of BCR in patients who received Mast + NoRT was significantly lower (8.25%; p < 0.001) (**Figure 1**). On the contrary, the 30-year CI of BSD was significantly higher in the Mast + NoRT group as compared with the Lum + RT group (21.28% vs. 36.72%; p < 0.001). CIF curves were plotted across patient subgroups to evaluate the impact of patients’ characteristics (**Figure S3**). Similar patterns of association between treatment modalities and incidences of BCR and BSD were observed (all p < 0.001).

### Competing risk for BCR and BSD

In univariate competing risk analysis, the incidence of recurrence was significantly greater in patients who received Lum + RT (HR: 1.900, 95% CI: 1.830–1.960, p < 0.001). After adjustment for covariates, the association between Lum + RT and higher incidence of BCR persisted (adjusted HR: 1.996, 95% CI: 1.925-2.069, p < 0.001) (**Table 3**). For competing risk analyses of BSD, Lum + RT was significantly associated with lower incidence of BSD before (HR: 0.408, 95% CI: 0.400-0.416, p < 0.001) and after (HR: 0.584, 95% CI: 0.572-0.597, p < 0.001) adjustment for covariates (**Table 3**).

**Table 3:** Risk of breast cancer recurrence (BCR) and breast cancer-specific death (BSD) and risk of breast cancer recurrence free survival (BRFS) and breast cancer-specific survival without recurrence (BCSS).

Competing Risk Models			Cox Proportional Hazards Models				
Outcomes	HR (95% CI)	*P* value	Outcomes	Before PSW		After PSW	
				HR (95% CI)	P value	HR (95%CI)	P value
**BCR**			**BRFS**				
Univariate	1.900 (1.830-1.960)	<0.001	Univariate	1.594 (1.530-1.661)	<0.001	1.824 (1.717-1.937)	<0.001
Multivariate^a^	1.996 (1.925-2.069)	<0.001	Multivariate^a^	1.788 (1.713-1.867)	<0.001	1.792 (1.716-1.871)	<0.001
**BSD**			**BCSS**				
Univariate	0.408 (0.400-0.416)	<0.001	Univariate	0.426 (0.416-0.436)	<0.001	0.675 (0.652-0.699)	<0.001
Multivariate^a^	0.584 (0.572-0.597)	<0.001	Multivariate^a^	0.699 (0.681-0.716)	<0.001	0.706 (0.688-0.725)	<0.001

HR, hazard ratio; CI, confidence interval; PSW, propensity score weighting.

a Multivariable analysis adjusted variables including age, race, year of diagnosis, historic subtype, tumor size, historic grade, stage, and chemotherapy.

### Survival analysis

**Figure 2:**
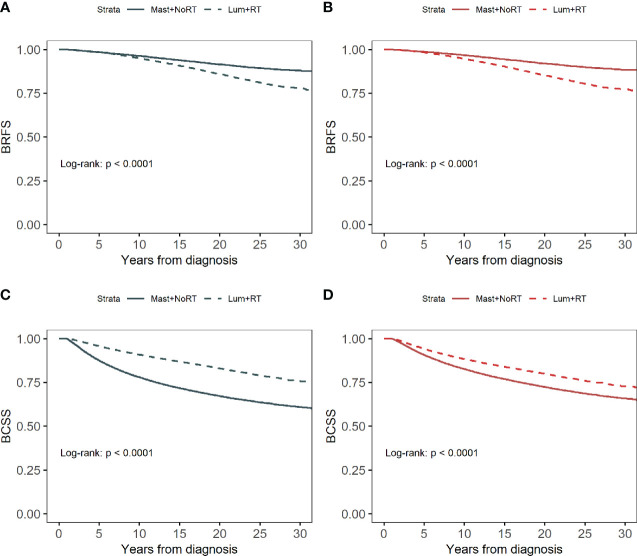
Kaplan–Meier curves for breast cancer recurrence-free survival (BRFS) by treatment before **(A)** and after **(B)** propensity score weighting. Kaplan–Meier curves for breast cancer-specific survival (BCSS) by treatment before **(C)** and after **(D)** propensity score weighting.

PSW-adjusted standardized differences were all less than 0.1 (**Figure S4**), indicating a good balance of covariates between the Lum + RT group and the Mast + NoRT group. The 30-year survival probability for BRFS estimated by the Kaplan-Meier method was 82.8%. The 30-year survival probability for BCSS was 69.1%. Lum + RT was associated with a significantly worse BRFS (log-rank p < 0.001 before and after PSW; **Figures 2A, B**), and a significant better BCSS when compared with Mast + NoRT (log-rank p <0.001 before and after PSW; **Figures 2C, D**).

**Table 3** shows the unadjusted and adjusted HRs and CIs for BRFS and BCSS estimated from Cox regression models. Patients in the Lum + RT group had significantly worse BRFS (adjusted HRs before/after PSW: 1.788 [95% CI 1.713-1.867] and 1.792 [95% CI 1.716-1.871], respectively). In contrast, Lum + RT was associated with better BCSS when compared with Mast + NoRT (adjusted HRs before/after PSW: 0.699 [95% CI 0.681-0.716] and 0.706 [95% CI 0.688-0.725], respectively). Detailed HRs and 95% CIs for Cox model covariates have been provided in **Table S1** and **Table S2**.

### Sensitivity analyses and stratified analyses

Competing risk regression analyses and Cox regression analyses in patients with different ER/PR statuses yielded similar results. Patients in the Lum + RT group had worse BRFS and better BCSS independent of ER/PR statuses (**Table S3**). Stratified analyses with PSW adjustment were carried out to assess whether treatment differences in survival outcomes depend on certain characteristics of patients. The weighted HRs were all < 1, showing an benefit for the Mast + NoRT treatment in these patient groups (**Figure S5A**). On the contrary, the weighted HRs for BCSS were all > 1, indicating a worse breast cancer-specific survival for patients who received Mast + NoRT (**Figure S5B**).

## Discussion

The use of lumpectomy plus adjuvant radiation had increased since the 1990s in our data, consistent with previous reports (2–4). We also noticed different trends by age. Lum + RT initially raised in the 2000s and then declined in the 2010s for women aged 18 to 44 years. A reverse trend was observed for mastectomy. For women aged over 60 years, mastectomy had been falling and Lum + RT had been increasing since the 1990s (data not shown). These trends were similar to a recent report (4).

In the early 1980s, large randomized controlled trials demonstrated that BCT provided long-term survival rates equivalent to that obtained after mastectomy for patients with early-stage invasive breast cancer (18–20). However, in agreement with previous studies (21–25, 44–51), our results observed long-term survival benefits in patients receiving Lum + RT compared to those receiving mastectomy. A previous study, which used SEER registry data on 83,776 women with breast cancer diagnosed between 1988 and 1997, found the best survival rates with combined lumpectomy and radiation (44). Our findings confirmed the superiority of lumpectomy plus adjuvant radiation to mastectomy alone using the updated SEER data, indicating that the survival benefit associated with Lum + RT had not changed over time. This finding was consistent across multiple analyses and all the predefined risk factors in our data. A registry-based study also demonstrated that women treated with primary mastectomy had a hazard ratio of 1.64 (95% CI 1.43-1.88) for breast cancer death compared with women treated with primary Lum + RT after adjusting for the year of diagnosis, age at diagnosis, stage, histology, and grade (47). Some had claimed that higher death rates in women treated with mastectomy was due to unfavorable prognostic patient characteristics such as preexisting comorbidities or older age (52). But this conflicted with the fact that elderly patients received more BCT treatment than mastectomy in recent years, which was also seen in our data. Also, a study conducted in Swedish national data from 48,986 breast cancer patients demonstrated that Lum+RT yielded better survival than mastectomy irrespective of RT despite adjustment for covariates including comorbidity burden (50). Lum + RT was associated with better overall survival and BCSS even in patients with triple-negative breast cancer tumors, which were generally more aggressive and associated with a worse prognosis (51).

Our results showed that patients receiving lumpectomy plus adjuvant radiation had a higher risk of locoregional recurrences than those receiving mastectomy alone. However, previous studies yielded inconsistent findings (13–19, 53, 54). Early trials suggested no significant difference in locoregional recurrence rates with BCT compared with mastectomy (15–17). Some studies found lower recurrence rates in patients who received BCT (18, 53, 54), yet others demonstrated similar findings to ours (13, 14, 19). One possible hypothesis for explaining higher recurrence rates with BCT is multifocality and multicentricity in young breast cancer patients, who constitute most of the patients treated with BCT (19). Nevertheless, based on previous researches, local recurrence in BCT did not seem to lead to worse survival (55, 56). Recurrence in the BCT was characterized by longer disease-free interval and related to a better prognosis (56). Our results also confirmed late recurrence in the Lum + RT group (**Figure S2B**). Thus, we believed that higher recurrence rates would not offset the survival advantage of BCT.

We acknowledge several limitations in the present study. The SEER registry dose not collect clinical data such as coexisting comorbidities, which may have influenced the treatment choice. Furthermore, the excess burden of comorbidities is related to shortened life expectancy. Nevertheless, this factor alone could not account for worse survival for patients who underwent mastectomy after adjusting for age and tumor characteristics. In addition, the current study lacks information on the details of treatments such as radiation dose, chemotherapy regimens, endocrine therapy and the specifics of radiotherapy, including dose, fields and type of radiation. Another concern has been the migration between SEER geographic area, which may lead to a loss of follow-up and an underestimation of the incidence of locoregional recurrences of breast cancer.

Our study has several strengths. To our knowledge, the present study is the first to examine oncologic outcomes between BCT and mastectomy in women with early-stage breast cancer from the population-based SEER registry by modern competing risk techniques. Competing risks arise when individuals are exposed to many causes of failure, and the occurrence of one failure hinders the occurrence of other failure events. Traditional survival analysis techniques such as the Kaplan-Meier curve and the Cox proportional hazard model treat failures from competing risks as censored, which may lead to overestimating the probability of outcomes of interest and biased results (30, 31, 57). The competing risk analyses and Cox proportional hazard model analyses yielded similar results in the current study, indicating a consistent independent association between BCT and better cancer-specific survival outcome. Moreover, we estimated long-term treatment-associated outcomes using data over the past 30 years. Risk estimation from short-term follow-up might be biased when a time-dependent risk factor is present. In addition, we applied the PSW method to adjust confounding factors between different treatment groups. The OW approach allows one to analyze an observational study to mimic a randomized experiment by modeling the assignment process of participants (58, 59).

## Conclusions

The analysis of the SEER registry data over the past 30 years (1988–2018) indicated that lumpectomy plus adjuvant radiation was associated with superior long-term breast cancer-specific survival and a higher risk of breast cancer recurrence when compared to mastectomy alone, independent of age, race, time period, historic subtype, tumor size, historic grade and stage. Because the survival advantage of Lum + RT cannot be explained by heterogeneity in patient characteristics, it may result from the treatment itself.

## Data availability statement

The datasets generated and/or analyzed during the present study are available in the SEER repository (https://seer.cancer.gov/).

## Ethics statement

The studies involving human participants were reviewed and approved by SEER. The ethics committee waived the requirement of written informed consent for participation. Written informed consent was not obtained from the individual(s) for the publication of any potentially identifiable images or data included in this article.

## Author contributions

SK: Study conceptualization, design and manuscript writing. WW: Data analysis, and manuscript editing. BL and XF: Data analysis, and manuscript editing. DY: Data analysis: JL: Study conceptualization, design, supervision, and manuscript writing. All authors contributed to the article and approved the submitted version.
