# Supplementary material for: Superior survival for breast-conserving therapy over mastectomy in patients with breast cancer: A population-based SEER database analysis across 30 years
Source: Front Oncol. 2023 Jan 4;12:1032063. doi: 10.3389/fonc.2022.1032063 (PMC9846313; doi:10.3389/fonc.2022.1032063)
Supplement: Supplementary file 1 [file Image_1.pdf]

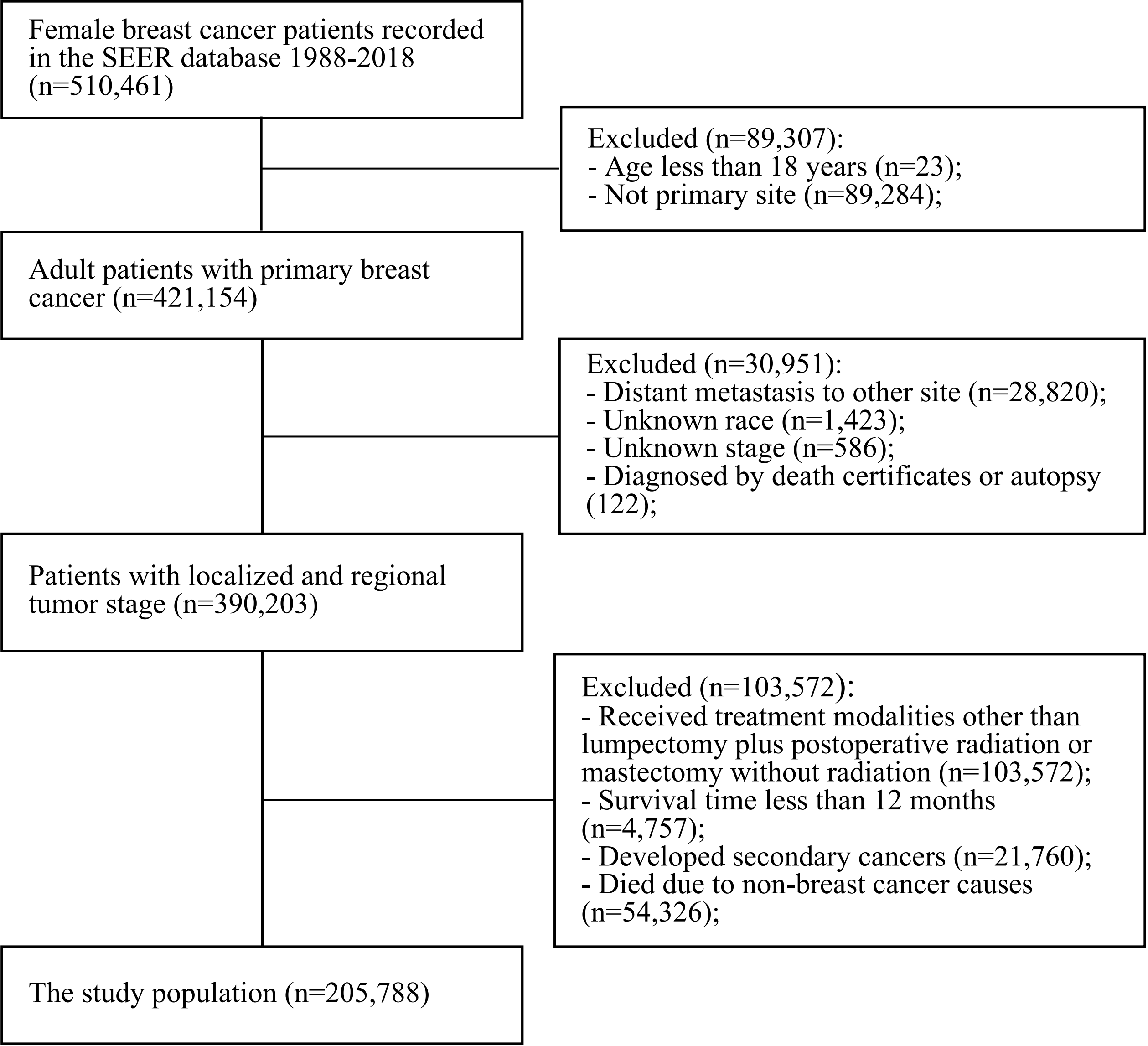
Female breast cancer patients recorded in the SEER database 1988-2018 (n=510,461)
Excluded (n=89,307):
- Age less than 18 years (n=23);
- Not primary site (n=89,284);
Adult patients with primary breast cancer (n=421,154)
Excluded (n=30,951):
- Distant metastasis to other site (n=28,820);
- Unknown race (n=1,423);
- Unknown stage (n=586);
- Diagnosed by death certificates or autopsy (122);
Patients with localized and regional tumor stage (n=390,203)
Excluded (n=103,572):
- Received treatment modalities other than lumpectomy plus postoperative radiation or mastectomy without radiation (n=103,572);
- Survival time less than 12 months (n=4,757);
- Developed secondary cancers (n=21,760);
- Died due to non-breast cancer causes (n=54,326);
The study population (n=205,788)
