# Supplementary figures and images for: Superior survival for breast-conserving therapy over mastectomy in patients with breast cancer: A population-based SEER database analysis across 30 years
Source: Front Oncol. 2023 Jan 4;12:1032063. doi: 10.3389/fonc.2022.1032063 (PMC9846313; doi:10.3389/fonc.2022.1032063)

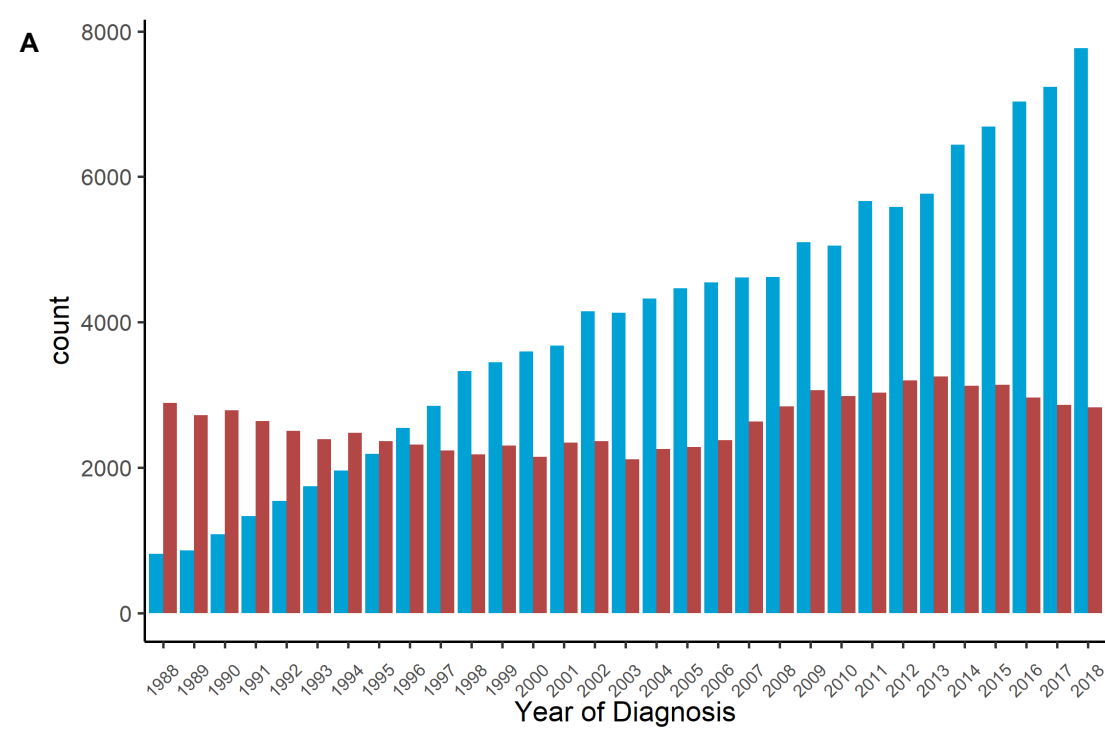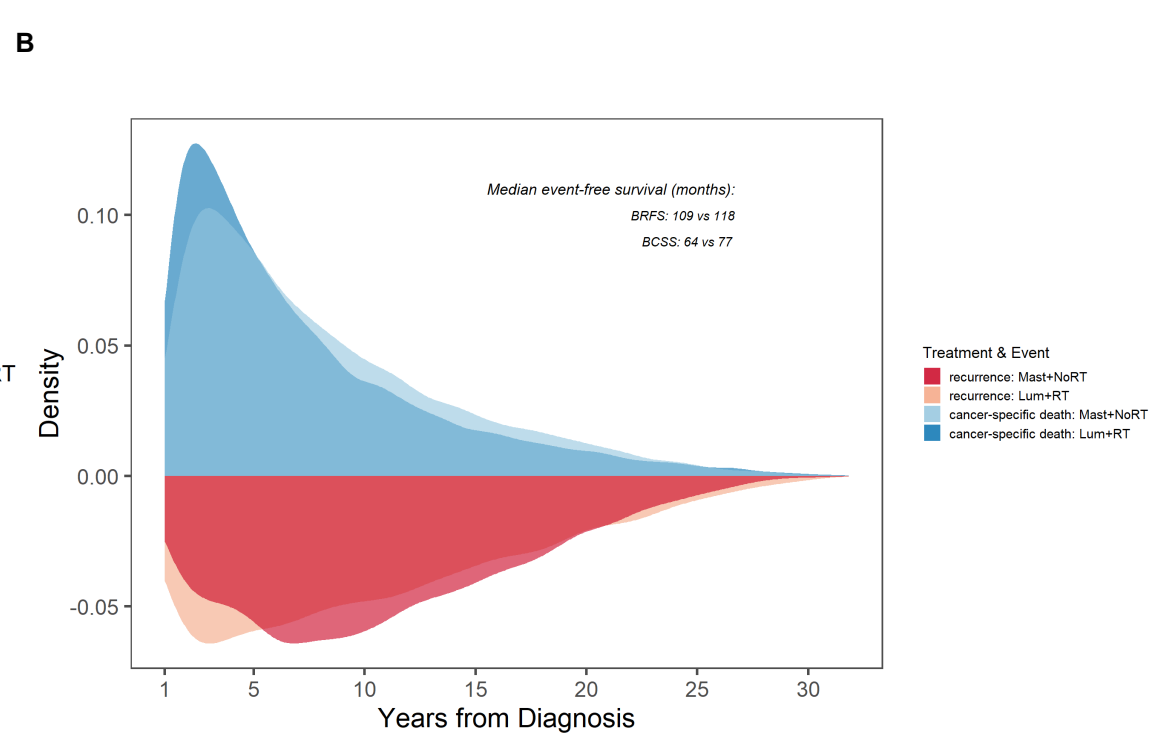

Supplement: Supplementary file 2 [file Image_2.pdf]

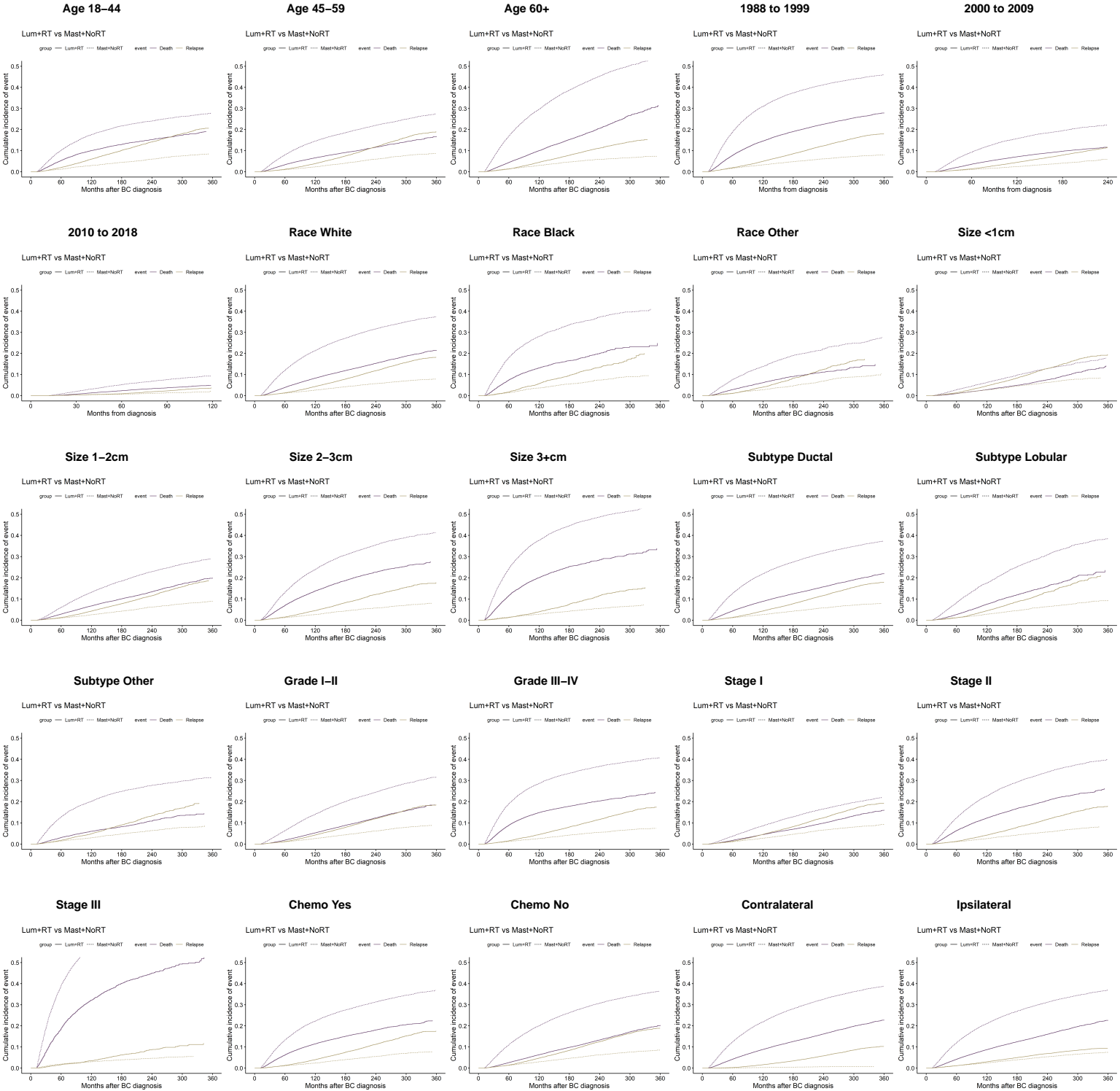

Supplement: Supplementary file 3 [file Image_3.pdf]

# Covariate Balance

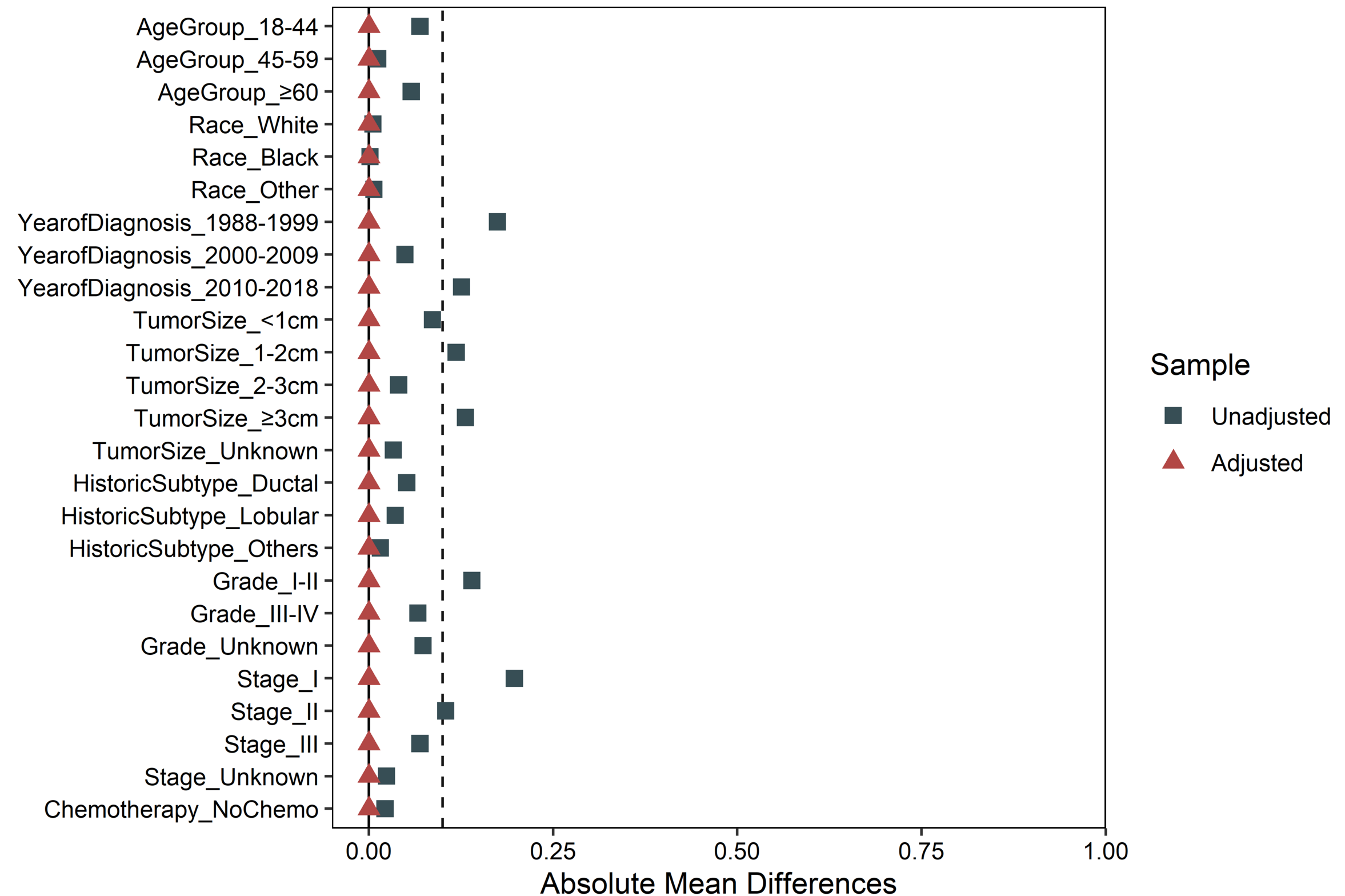

Supplement: Supplementary file 4 [file Image_4.pdf]

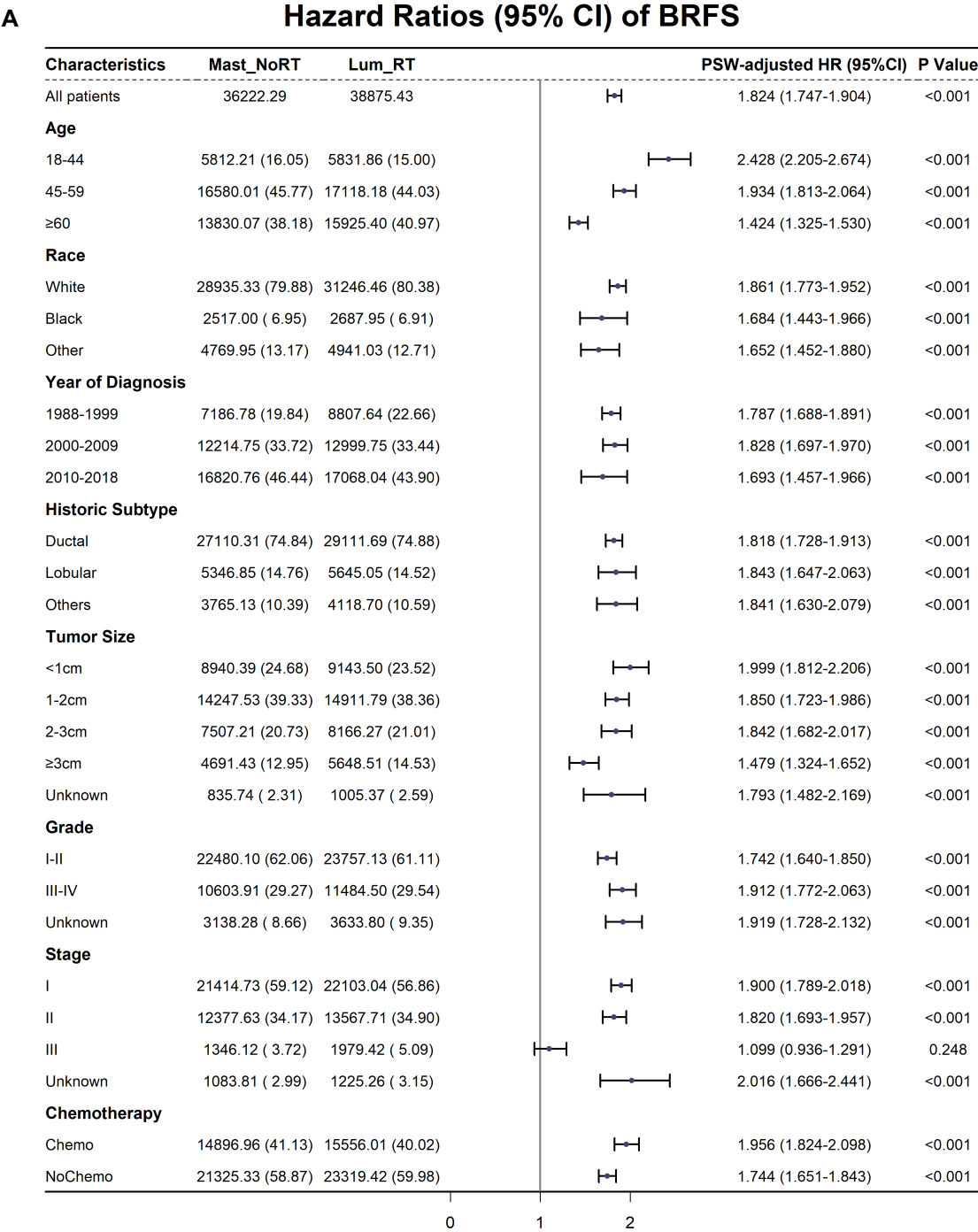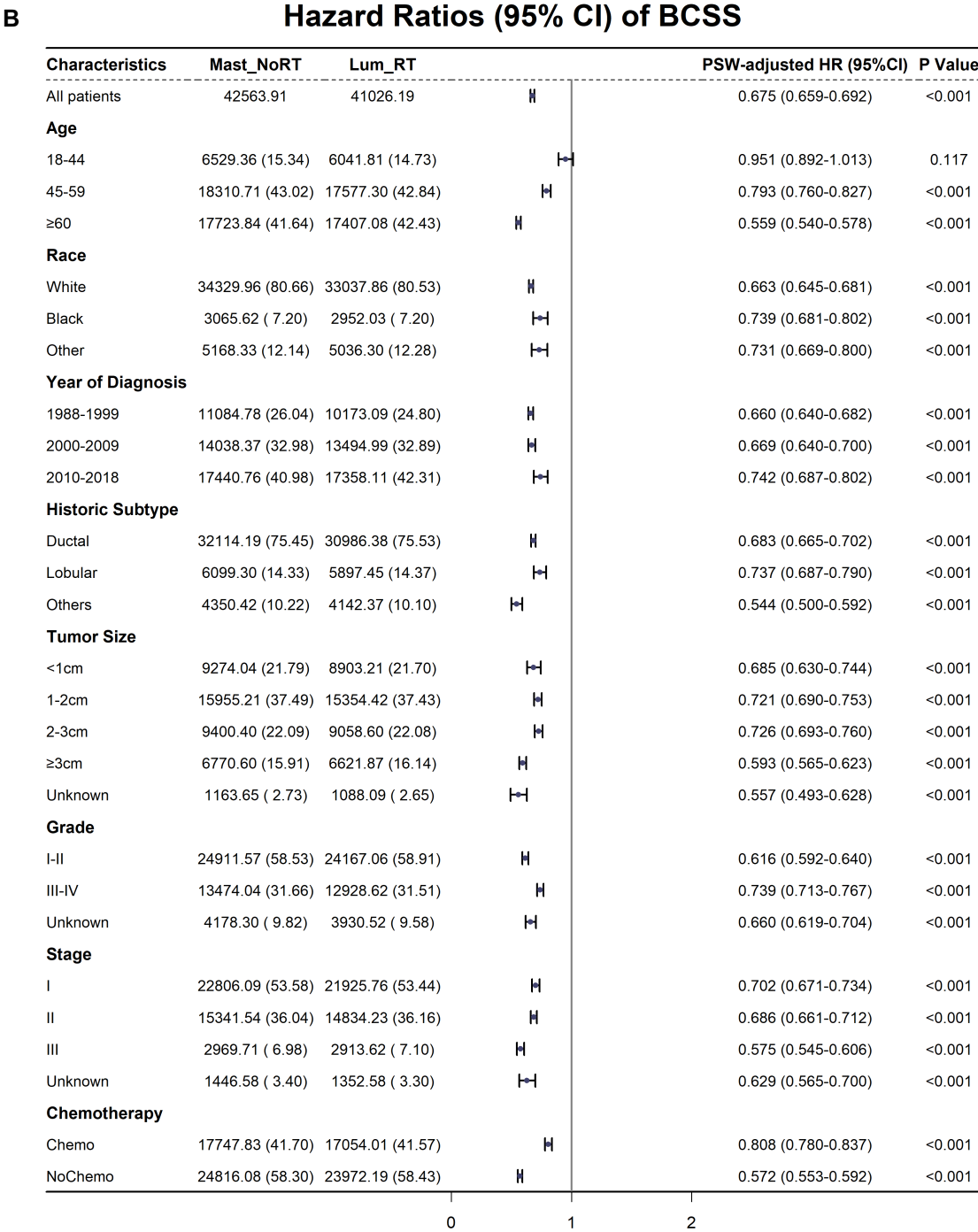

Supplement: Supplementary file 5 [file Image_5.pdf]
