# Supplementary material for: Superior survival for breast-conserving therapy over mastectomy in patients with breast cancer: A population-based SEER database analysis across 30 years
Source: Front Oncol. 2023 Jan 4;12:1032063. doi: 10.3389/fonc.2022.1032063 (PMC9846313; doi:10.3389/fonc.2022.1032063)
Supplement: Supplementary file 6 [file DataSheet_1.docx]

**Table S1. Univariate and multivariate Cox proportional hazard models for breast cancer recurrence-free survival (BRFS) before and after propensity score weighting (PSW).**

| **Factors** | **Before PSW** | | | | |  | **After PSW** | | | | |
| --- | --- | --- | --- | --- | --- | --- | --- | --- | --- | --- | --- |
|  | **Univariate model** | |  | **Multivariate model^a^** | |  | **Univariate model** | |  | **Multivariate model^a^** | |
|  | **HR (95% CI)** | ***P* value** |  | **HR (95% CI)** | ***P* value** |  | **HR (95% CI)** | ***P* value** |  | **HR (95% CI)** | ***P* value** |
| **Treatment Mast + NoRT** | 1.594 (1.530-1.661) | <0.001 |  | 1.788 (1.713-1.867) | <0.001 |  | 1.824 (1.717-1.937) | <0.001 |  | 1.792 (1.716-1.871) | <0.001 |
| **Age Group 45-60** | 0.845 (0.803-0.890) | <0.001 |  | 0.851 (0.808-0.896) | <0.001 |  | 0.794 (0.737-0.856) | <0.001 |  | 0.836 (0.791-0.883) | <0.001 |
| **Age Group ≥60** | 0.993 (0.942-1.048) | 0.811 |  | 1.041 (0.983-1.102) | 0.170 |  | 0.922 (0.851-0.998) | <0.001 |  | 1.021 (0.961-1.084) | 0.508 |
| **Race Black** | 1.329 (1.238-1.426) | <0.001 |  | 1.324 (1.233-1.422) | <0.001 |  | 1.343 (1.207-1.494) | <0.001 |  | 1.332 (1.234-1.438) | <0.001 |
| **Race Other** | 0.962 (0.904-1.024) | 0.221 |  | 1.007 (0.947-1.072) | 0.814 |  | 0.985 (0.898-1.080) | 0.653 |  | 1.024 (0.959-1.094) | 0.475 |
| **Year of Diagnosis 2000-2009** | 0.752 (0.720-0.785) | <0.001 |  | 0.705 (0.674-0.738) | <0.001 |  | 0.697 (0.653-0.745) | <0.001 |  | 0.712 (0.679-0.747) | <0.001 |
| **Year of Diagnosis 2010-2018** | 0.485 (0.450-0.522) | <0.001 |  | 0.439 (0.406-0.474) | <0.001 |  | 0.437 (0.388-0.491) | <0.001 |  | 0.441 (0.405-0.480) | <0.001 |
| **Historic Subtype Lobular** | 1.037 (0.981-1.096) | 0.197 |  | 1.100 (1.039-1.165) | <0.001 |  | 1.065 (0.981-1.158) | 0.033 |  | 1.104 (1.039-1.172) | <0.001 |
| **Historic Subtype Others** | 1.053 (0.994-1.116) | 0.077 |  | 1.031 (0.972-1.093) | 0.313 |  | 1.088 (0.998-1.187) | <0.001 |  | 1.036 (0.973-1.104) | 0.264 |
| **Tumor Size 1-2cm** | 1.009 (0.960-1.060) | 0.725 |  | 1.021 (0.971-1.075) | 0.413 |  | 1.014 (0.939-1.095) | 0.605 |  | 1.022 (0.969-1.078) | 0.428 |
| **Tumor Size 2-3cm** | 1.099 (1.038-1.164) | <0.001 |  | 1.168 (1.089-1.252) | <0.001 |  | 1.165 (1.069-1.269) | <0.001 |  | 1.177 (1.095-1.265) | <0.001 |
| **Tumor Size ≥3cm** | 1.114 (1.043-1.190) | <0.001 |  | 1.233 (1.133-1.342) | <0.001 |  | 1.204 (1.089-1.33) | <0.001 |  | 1.206 (1.104-1.318) | <0.001 |
| **Tumor Size Unknown** | 1.071 (0.969-1.184) | 0.177 |  | 1.289 (1.110-1.497) | <0.001 |  | 1.238 (1.067-1.436) | <0.001 |  | 1.252 (1.055-1.484) | <0.001 |
| **Grade III-IV** | 1.081 (1.036-1.127) | <0.001 |  | 1.084 (1.035-1.134) | <0.001 |  | 1.115 (1.046-1.188) | <0.001 |  | 1.070 (1.020-1.123) | <0.001 |
| **Grade Unknown** | 1.150 (1.087-1.218) | <0.001 |  | 1.091 (1.027-1.158) | <0.001 |  | 1.264 (1.163-1.374) | <0.001 |  | 1.092 (1.026-1.163) | <0.001 |
| **Stage II** | 1.001 (0.960-1.043) | 0.959 |  | 1.029 (0.970-1.090) | 0.343 |  | 1.060 (0.997-1.128) | <0.001 |  | 1.032 (0.972-1.095) | 0.310 |
| **Stage III** | 1.366 (1.262-1.479) | <0.001 |  | 1.431 (1.305-1.569) | <0.001 |  | 1.403 (1.241-1.584) | <0.001 |  | 1.336 (1.21-1.476) | <0.001 |
| **Stage Unknown** | 0.981 (0.893-1.077) | 0.685 |  | 0.823 (0.713-0.950) | <0.001 |  | 1.122 (0.979-1.286) | 0.025 |  | 0.858 (0.731-1.008) | 0.063 |
| **Chemotherapy Chemo** | 0.926 (0.891-0.963) | <0.001 |  | 0.838 (0.800-0.878) | <0.001 |  | 0.930 (0.877-0.985) | <0.001 |  | 0.842 (0.801-0.885) | <0.001 |

HR: hazard ratio; CI: confidence interval.

a Multivariable Cox proportional hazards analysis adjusted variables including age, race, year of diagnosis, historic subtype, tumor size, historic grade, stage, and chemotherapy.

**Table S2. Univariate and multivariate Cox proportional hazard models for breast cancer-specific survival (BCSS) before and after propensity score weighting (PSW).**

| **Factors** | **Before PSW** | | | | |  | **After PSW** | | | | |
| --- | --- | --- | --- | --- | --- | --- | --- | --- | --- | --- | --- |
|  | **Univariate model** | |  | **Multivariate model^a^** | |  | **Univariate model** | |  | **Multivariate model^a^** | |
|  | **HR (95% CI)** | ***P* value** |  | **HR (95% CI)** | ***P* value** |  | **HR (95% CI)** | ***P* value** |  | **HR (95% CI)** | ***P* value** |
| **Treatment Mast + NoRT** | 0.426 (0.416-0.436) | <0.001 |  | 0.699 (0.681-0.716) | <0.001 |  | 0.675 (0.652-0.699) | <0.001 |  | 0.706 (0.688-0.725) | <0.001 |
| **Age Group 45-60** | 0.753 (0.727-0.78) | <0.001 |  | 1.030 (0.994-1.067) | 0.100 |  | 0.815 (0.772-0.859) | <0.001 |  | 0.993 (0.954-1.034) | 0.737 |
| **Age Group ≥60** | 1.506 (1.457-1.557) | <0.001 |  | 2.337 (2.257-2.419) | <0.001 |  | 1.692 (1.609-1.780) | <0.001 |  | 2.269 (2.183-2.359) | <0.001 |
| **Race Black** | 1.396 (1.342-1.453) | <0.001 |  | 1.361 (1.307-1.416) | <0.001 |  | 1.414 (1.332-1.501) | <0.001 |  | 1.347 (1.286-1.410) | <0.001 |
| **Race Other** | 0.683 (0.654-0.713) | <0.001 |  | 0.753 (0.722-0.786) | <0.001 |  | 0.667 (0.626-0.712) | <0.001 |  | 0.752 (0.718-0.788) | <0.001 |
| **Year of Diagnosis 2000-2009** | 0.385 (0.375-0.395) | <0.001 |  | 0.494 (0.480-0.508) | <0.001 |  | 0.453 (0.436-0.471) | <0.001 |  | 0.490 (0.476-0.505) | <0.001 |
| **Year of Diagnosis 2010-2018** | 0.212 (0.204-0.221) | <0.001 |  | 0.280 (0.268-0.292) | <0.001 |  | 0.262 (0.246-0.278) | <0.001 |  | 0.281 (0.269-0.294) | <0.001 |
| **Historic Subtype Lobular** | 0.958 (0.926-0.990) | 0.011 |  | 0.849 (0.821-0.879) | <0.001 |  | 0.864 (0.821-0.910) | <0.001 |  | 0.862 (0.831-0.893) | <0.001 |
| **Historic Subtype Others** | 0.846 (0.814-0.879) | <0.001 |  | 0.802 (0.771-0.834) | <0.001 |  | 0.788 (0.743-0.836) | <0.001 |  | 0.787 (0.754-0.821) | <0.001 |
| **Tumor Size 1-2cm** | 2.051 (1.959-2.147) | <0.001 |  | 1.681 (1.604-1.761) | <0.001 |  | 2.000 (1.871-2.137) | <0.001 |  | 1.665 (1.587-1.747) | <0.001 |
| **Tumor Size 2-3cm** | 4.067 (3.884-4.258) | <0.001 |  | 2.129 (2.021-2.243) | <0.001 |  | 3.610 (3.377-3.860) | <0.001 |  | 2.120 (2.007-2.238) | <0.001 |
| **Tumor Size ≥3cm** | 7.124 (6.809-7.454) | <0.001 |  | 2.643 (2.504-2.789) | <0.001 |  | 5.601 (5.238-5.990) | <0.001 |  | 2.673 (2.525-2.831) | <0.001 |
| **Tumor Size Unknown** | 4.741 (4.457-5.044) | <0.001 |  | 1.806 (1.665-1.961) | <0.001 |  | 3.612 (3.274-3.985) | <0.001 |  | 1.600 (1.450-1.766) | <0.001 |
| **Grade III-IV** | 2.269 (2.213-2.326) | <0.001 |  | 1.582 (1.541-1.624) | <0.001 |  | 2.037 (1.963-2.114) | <0.001 |  | 1.581 (1.537-1.626) | <0.001 |
| **Grade Unknown** | 2.346 (2.270-2.424) | <0.001 |  | 1.356 (1.310-1.404) | <0.001 |  | 1.920 (1.824-2.021) | <0.001 |  | 1.376 (1.326-1.427) | <0.001 |
| **Stage II** | 2.699 (2.625-2.775) | <0.001 |  | 1.751 (1.688-1.816) | <0.001 |  | 2.392 (2.296-2.491) | <0.001 |  | 1.746 (1.681-1.814) | <0.001 |
| **Stage III** | 8.900 (8.626-9.184) | <0.001 |  | 4.518 (4.335-4.709) | <0.001 |  | 7.181 (6.841-7.539) | <0.001 |  | 4.548 (4.348-4.758) | <0.001 |
| **Stage Unknown** | 2.879 (2.731-3.034) | <0.001 |  | 1.704 (1.580-1.837) | <0.001 |  | 2.569 (2.372-2.783) | <0.001 |  | 1.879 (1.720-2.054) | <0.001 |
| **Chemotherapy Chemo** | 1.247 (1.219-1.275) | <0.001 |  | 0.876 (0.853-0.899) | <0.001 |  | 1.243 (1.201-1.286) | <0.001 |  | 0.843 (0.819-0.867) | <0.001 |

HR: hazard ratio; CI: confidence interval.

a Multivariable Cox proportional hazards analysis adjusted variables including age, race, year of diagnosis, historic subtype, tumor size, historic grade, stage, and chemotherapy.

**Table S3. Risk of breast cancer recurrence (BCR), breast cancer-specific death (BSD), breast cancer recurrence free survival (BRFS), and breast cancer-specific survival (BCSS) in patients with different ER/PR statuses.**

| **Competing Risk Models** | | |  | **Cox Proportional Hazards Models** | | |
| --- | --- | --- | --- | --- | --- | --- |
| **Outcomes** | **HR (95% CI)** | ***P* value** |  | **Outcomes** | **HR (95% CI)** | ***P* value** |
|  |  |  |  |  |  |  |
| **BCR** |  |  |  | **BRFS** |  |  |
| ER-/PR- | 2.21 (2.03-2.41) | <0.001 |  | ER-/PR- | 1.90 (2.10-1.71) | <0.001 |
| ER-/PR+ | 1.94 (1.57-2.39) | <0.001 |  | ER-/PR+ | 1.55 (1.99-1.20) | <0.001 |
| ER+/PR- | 2.05 (1.81-2.31) | <0.001 |  | ER+/PR- | 1.69 (1.95-1.46) | <0.001 |
| ER+/PR+ | 1.82 (1.73-1.91) | <0.001 |  | ER+/PR+ | 1.60 (1.69-1.51) | <0.001 |
| **BSD** |  |  |  | **BCSS** |  |  |
| ER-/PR- | 0.52 (0.50-0.55) | <0.001 |  | ER-/PR- | 0.55 (0.58-0.52) | <0.001 |
| ER-/PR+ | 0.42 (0.36-0.47) | <0.001 |  | ER-/PR+ | 0.44 (0.51-0.37) | <0.001 |
| ER+/PR- | 0.41 (0.39-0.44) | <0.001 |  | ER+/PR- | 0.43 (0.46-0.40) | <0.001 |
| ER+/PR+ | 0.43 (0.41-0.44) | <0.001 |  | ER+/PR+ | 0.44 (0.46-0.43) | <0.001 |

HR: hazard ratio; CI: confidence interval;
